# The role of the microbiome in allergic dermatitis-related otitis externa: a multi-species comparative review

**DOI:** 10.3389/fvets.2024.1413684

**Published:** 2024-12-16

**Authors:** Cyrelle Houtsaeger, Frank Pasmans, Ingmar Claes, Sophie Vandenabeele, Freddy Haesebrouck, Sarah Lebeer, Filip Boyen

**Affiliations:** ^1^Department of Pathobiology Pharmacology and Zoological Medicine, Faculty of Veterinary Medicine, Ghent University, Merelbeke, Belgium; ^2^YUN NV, Niel, Belgium; ^3^Department of Bioscience Engineering, University of Antwerp, Antwerp, Belgium; ^4^Department of Small Animal Medicine and Clinical Biology, Faculty of Veterinary Medicine, Ghent University, Merelbeke, Belgium

**Keywords:** allergic dermatitis, microbiome, otitis externa, ear microbiome, atopic dermatitis, external ear canal

## Abstract

The external ear canal, characterized by species-specific structural and physiological differences, maintains a hostile environment that prevents microbial overgrowth and foreign body entry, supported by factors such as temperature, pH, humidity, and cerumen with antimicrobial properties. This review combines several studies on the healthy ear canal’s structure and physiology with a critical approach to the potential existence of an ear microbiome. We use a comparative multi-species approach to explore how allergic conditions alter the ear canal microenvironment and cerumen in different mammalian species, promoting pathogen colonization. We propose a pathogenetic model in which allergic conditions disrupt the antimicrobial environment of the EEC, creating circumstances favorable for facultative pathogenic micro-organisms like *Staphylococcus* and *Malassezia* species, leading to otitis externa (OE). A better understanding of the underpinning mechanisms may lead to innovative approaches to disease mitigation.

## 1 Introduction

Inflammation of the external ear canal (EEC), otitis externa (OE), is a common dermatological disorder, diagnosed in approximately 1% of human patients in primary care (1). Changes in parameters such as the pH, temperature, or composition of earwax—a unique waxy substance derived from desquamated epithelial cells, sebaceous and ceruminous glands—can create an ideal environment for bacteria and/or yeasts to colonize and infect the ear canal. The OE-patient may present with pain, erythema and pruritis of the EEC and when microbial overgrowth is confirmed by cytology, topical antimicrobial treatment is required (2, 3). Complications include otitis media, otitis interna and even osteomyelitis of the temporal bone (2, 4, 5). Importantly, as long as the predisposing factor for OE—such as regular swimming, local trauma or allergic disease (6–8)—is not removed, these patients are at high risk of recurrence, resulting in chronic OE which often requires repeated antimicrobial treatment.

Antimicrobial resistance in ear pathogens is increasing (9, 10), which necessitates the development of alternatives to treat and prevent OE. As has been proposed in other skin disorders such as acne vulgaris and allergic dermatitis (11–13), it may be relevant to study new strategies that—instead of limiting the growth of ear pathogens by using bacteriostatic and bacteriocidic drugs—help to restore the balance between commensal microorganisms in the EEC, referred to as the ear microbiome. However—until now—little evidence exists on the presence, complexity, and protective role of a potential microbial community within the EEC. Moreover, it appears that the EEC is provided with many features that limit the growth of microorganisms, as will be discussed in this review.

We here aimed at investigating the presence and potential role of an ear microbiome by identifying the different challenges encountered by microorganisms entering the EEC, such as the different components of cerumen and the acidic pH. We focus on the disturbing impact of allergic dermatitis (AD)—a common underlying factor for OE—on different components of the microenvironment of the EEC to identify potential mechanisms favoring pathogen overgrowth. We use a cross-species comparative approach to identify common drivers of OE by including humans, dogs (prevalence of allergic OE comparable to humans) and horses (low allergic OE prevalence). By identifying the species-specific factors that contribute to maintaining a hostile environment within the ear canal, we aim to investigate the presence of an ear microbiome through a rigorous and critical analysis. Considering the impact of allergic dermatitis on the EEC and cerumen, we tried to identify new leads for future research into the prevention and treatment of allergy-induced OE.

## 2 The EEC in health and allergic dermatitis

An overview of the features of OE and the pathogenesis of allergic dermatitis related to OE in humans, dogs and horses is given in Table 1 and Supplementary Table 1. AD refers to different forms of AD, including a local hypersensitivity inflammatory response after exposure such as allergic contact dermatitis (14), and atopic dermatitis, a generalized chronic immune-mediated inflammatory skin disorder associated with IgE-antibodies (15–17). As shown in Table 1, OE in humans is associated with a local inflammatory reaction that occurs after direct contact of the EEC with hearing aids or chemicals in cosmetic products, while OE in dogs is associated with a generalized inflammatory reaction to environmental allergens (atopic dermatitis). The pathogenesis of atopic dermatitis in humans and dogs shows many similarities (18), however—as opposed to dogs with atopy (19)—OE is not a typical symptom of atopic dermatitis in humans (19). The question arises why this form of AD seems to have less impact on the human EEC compared with the canine EEC.

**Table 1:** Prevalence of (allergic-dermatitis-related) OE in humans, dogs and horses and features of the allergic disease most associated with OE.

	Human	Dog	Horse
Prevalence of OE	Up to 1% (1)	7.3-10% (142–144)	Unknown (5, 26)
Common bacteria/ yeast/ fungi associated with OE	*Pseudomonas aeruginosa;* *Staphylococcus aureus;* *Staphylococcus epidermidis;* *Cutibacterium acnes;* *Corynebacterium jeikeium* *Candida* species and *Aspergillus* species (9, 110, 145–149).	*Malassezia pachydermatis;* *Staphylococcus pseudintermedius;* *Pseudomonas aeruginosa;* *Streptococcus* species; *Proteus mirabilis;* *Escherichia coli* and *Corynebacterium* species (100, 103, 105–107, 150).	*Enterococcus faecalis;* *Staphylococcus delphini;* *Staphylococcus aureus;* Coagulase-negative *Staphylococcus* species *Actinobacillus* species and *β*-haemolytic *Streptococcus* species (5).
Prevalence of allergic-dermatitis related OE	23-59% (7)	8% (19)	Unknown (5, 151, 152)
Most common type of allergic dermatitis associated with OE	Allergic contact dermatitis (7)	Atopic dermatitis (19, 153)	Insect-bite-hypersensitivity (5)
Extensiveness of the allergic reaction	Local reaction (154)	General reaction (17)	General reaction (155)
Origin of the allergens associated with the allergic disease	Hearing aids, ototopical products (7)	Environmental allergens (156)	Saliva from *Culicoides* insects (151)

### 2.1 Structure related to disease

The anatomy of the EEC differs greatly in humans, dogs and horses; both in length, shape and, most interesting, the ratio between the cartilaginous—containing sebaceous and ceruminous glands—and the non-glandular osseous parts of the ear canal. The comparison of the species-specific differences in the anatomic features and physiologic parameters of the EEC is shown in Table 2. The canine EEC is almost exclusively supported by cartilage and as such, entirely provided with sebaceous and ceruminous glands. Furthermore, the canine ear canal is defined by a site-dependent distribution of sebaceous and ceruminous glands, with an increasing ceruminous/sebaceous gland ratio toward the tympanum (20–23). In contrast, cartilage supports only 1/3rd to 1/4th of the human EEC and 3/4th of the equine EEC (24–28). It is possible that these anatomic varieties have a different impact on both the volume and composition of cerumen between these species. Structure-related predisposing factors for OE include both physical and histologic alterations. Narrowing of the EEC lumen—due to anatomical varieties, chronic changes as well as accumulation of cerumen—is associated with OE and ceruminoliths in humans and dogs (19, 29, 30). Reduced ear canal permeability might affect the antimicrobial microenvironment in the external ear, providing better conditions for pathogens to survive and colonize.

**Table 2:** Comparison of the anatomy, histology and physiology of the human, canine and equine EEC.

	Human	Dog	Horse
Anatomy of the EEC			
Shape and direction	Mild ‘S’ shape, horizontal (23)	Starting from the EEC opening: distal vertical and proximal horizontal canal (157)	Hourglass shape, vertical (26)
Length	2.5–3 cm (23)	Breed-specific, positively correlated with body weight (on average 5.3 ± 1 cm) (158)	Dorsal length of the osseous EEC: 2.51–3.08 cm (26)
Histology of the EEC			
Sebaceous glands	Only present in the cartilaginous part, most apparent in outer two-thirds of the EEC (30)	Only present in the cartilaginous part, most apparent toward external ear opening (157, 158)	Only present in the cartilaginous part (159)
Ceruminous glands	Only present in the cartilaginous part, most apparent in outer two-thirds of the EEC (30)	Only present in the cartilaginous part, most apparent toward tympanic membrane (157, 158)	Only present in the cartilaginous part (159)
Ratio cartilaginous: osseus EEC	1: 2 (30)	4: 1 (157, 158)	2.5: 1 (159)
Microenvironment			
Relative temperature	36.4 ± 0.6°C (37)	Erect ears: 37.17 ± 0.85°C (160) Semi-pendulous ears:37.35 ± 0.5°C (160) Pendulous ears: 36.93 ± 0.96°C (160)	No data available
pH	5.4 ± 0.48 (161)	4.6–7.2 (162)	No data available
Relative humidity	Left ear: 51.7 ± 9.6 (163) Right ear: 50.6 ± 9.5 (163)	Erect ears: 98.6 ± 1.0 (39) Semi-pendulous ears: 91.9 ± 3.7 (39) Pendulous ears: 93.1 ± 3.0 (39)	No data available

Allergic dermatitis is associated with a disruption and alteration of the skin barrier, which lines the EEC. Both in humans and dogs, atopic skin is characterized by a higher abundance of short chain ceramides compared to long chain ceramides, which impedes the integrity of the skin barrier and causes an increase in transepidermal water loss (31–34). Moreover, lesional skin from atopic dogs is defined by a decreased level of sphingomyelins and cholesteryl esters, essential compounds of the epidermal barrier (34). The expression of filaggrin, a crucial protein in the epidermal barrier; and claudin-1, an essential component of tight junctions, is decreased in human patients with atopic dermatitis, thereby further impairing the skin barrier (35, 36). The contribution of filaggrin mutations on the pathogenesis of CAD remains to be elucidated (18).

Considering the anatomic variations between dogs and humans, it seems possible that the inflammatory parameters associated with the pathogenesis of atopic dermatitis have a different impact on the canine EEC than on the human EEC, which could explain the difference in predisposition for OE in atopic patients of both species. The canine EEC is relatively longer than the human ear canal and in contrast to the simple, short horizontal EEC of humans, the canine EEC consists of a vertical and horizontal canal, with a sharp junction in between. Moreover, the canine EEC is almost completely covered by cerumen, which is in great contrast to humans, where sebaceous and ceruminous glands are only present in a small part of the EEC. Therefore, the induction of a general cutaneous inflammatory response in atopic subjects could hypothetically have a greater impact on the microenvironment and microbiome—containing facultative pathogens—in a longer, more enclosed, perhaps less aerobic canine EEC than on the shorter, more open human EEC.

Regarding the length of the EEC and the distribution of the sebaceous and ceruminous glands, the equine EEC can be considered as intermediate between the human and canine EEC. Interestingly, horses with Insect-bite-hypersensitivity (IBH), an allergic condition which resembles atopic dermatitis as discussed above, are also at risk for OE. This strengthens the assumption that the variations in length and distribution of the different glands in the EEC may, at least partly, be responsible for the different effect of allergies on the EEC in humans, dogs and horses.

### 2.2 (Patho) physiology of the EEC

An overview of the physiologic parameters of the EEC is shown in Table 2.

The temperature within the EEC resembles the average body temperature, both in dogs and humans. Yet, ear temperature was found to be significantly lower in men and elderly people, compared to women and adults, respectively (37). Also in dogs, ear temperature differed depending on age. Huang et al. demonstrated that dogs younger than 6 years and dogs weighing less than 6 kg showed significantly higher ear temperatures than dogs over 6 years old and over 6 kg bodyweight, respectively (38). So far, ear temperature has not been identified as a contributing factor to ear disease. Considering ‘calor’ as a key element in an inflammatory reaction, one could expect an increase in ear temperature during inflammation, which could potentially alter the growth and survival of certain microorganisms and stimulate an immunologic response. However, Yoshida et al. measured the EEC temperature in both OE and healthy dogs and did not find significant differences between these 2 groups of dogs (39).

As shown in Table 2, the pH of the human EEC is moderate to strong acidic, and as such, comparable to the skin pH. This low pH is caused by exocytosis of lysosomes and hydrolyzation of phospholipids, helps in creating an antimicrobial environment, enhances the epidermal barrier and has an influence on the epidermal lipid synthesis (40). Interestingly, the pH within the canine EEC appears to be less acidic, and also the skin pH in dogs, and in other mammals, is on average higher than the pH of human skin, which is possibly due to the presence of the hairy coat in mammals (e.g., skin pH in dogs: 6-7, skin pH in cats: 6.4–6.9, skin pH in horses: 7-8) (40). An acidic skin pH ensures better adhesion of the resident skin microbiota on the skin surface (41). Therefore, it is possible that the composition and diversity of the ear microbiome is affected differently by the acidic pH in the human ear canal and the higher pH in the canine ear canal.

The effect of allergic dermatitis on the otic pH, temperature and humidity has not been studied so far. In dogs, lesional atopic skin is determined by an elevation in skin pH (42). These changes in pH, however small, can have a significant influence on the microbiome, favoring the colonization of certain pathogens such as *Staphylococcus aureus* (43).

## 3 Cerumen as a defense mechanism toward OE-pathogens

Cerumen is a crucial biofluid in preventing microbial growth and infection of the EEC. To fulfill this function, cerumen contains several fundamental compounds, derived from desquamated epithelial cells and secretions of the sebaceous and ceruminous glands. In this section, the different compounds in cerumen with a defined antimicrobial action will be discussed.

### 3.1 The physical trapping function of cerumen: the contribution of lipids and mucins

#### 3.1.1 Lipids

**Figure 1:**
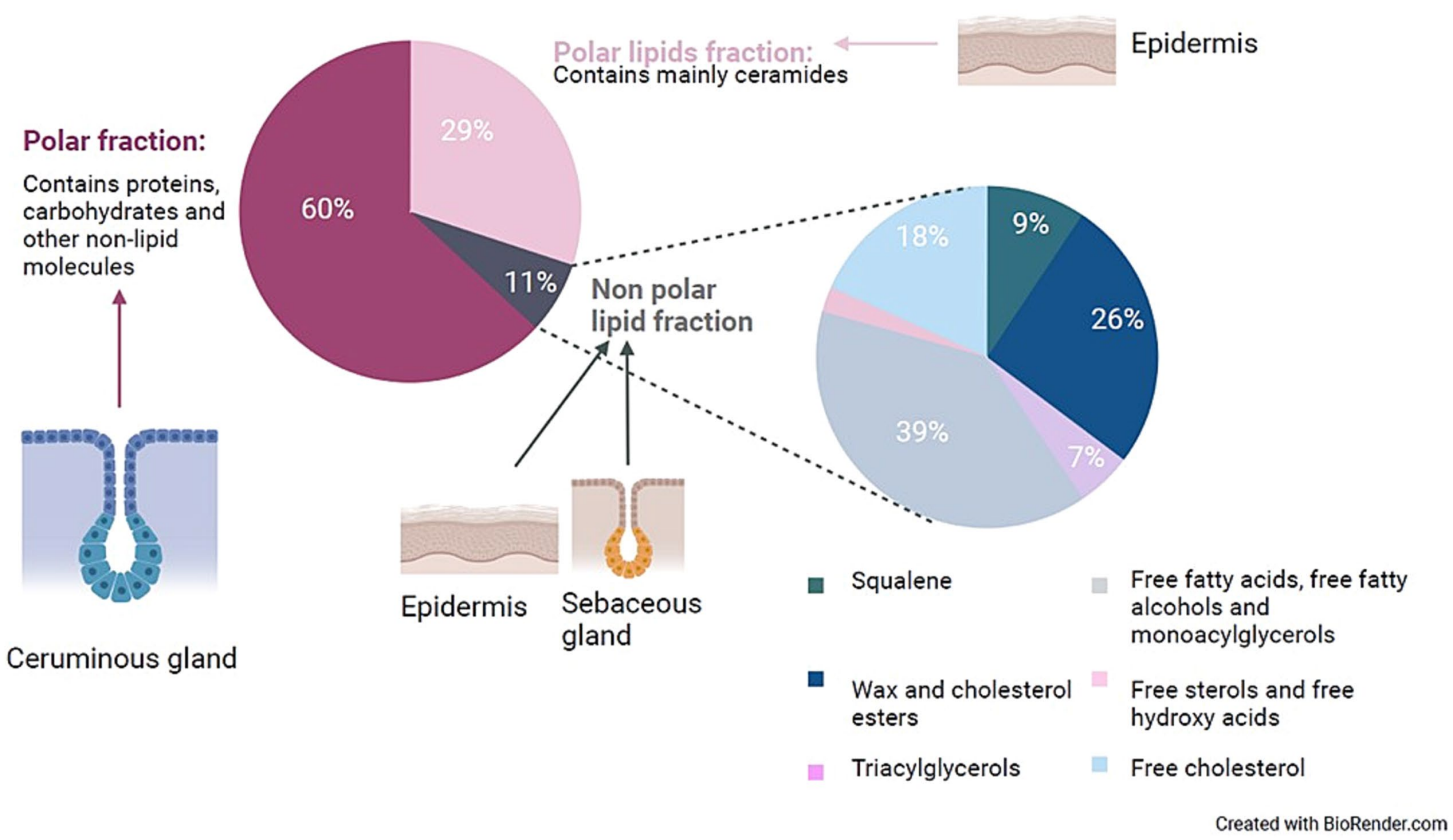
Presentation of the distribution and probable origin of the components of human cerumen. This figure was made by the authors of this review, based on the rough data available in the study by Stransky et al. (77) and the suggested origin of these lipids was based on literature research. During the study of Stransky et al., 200 doses of cerumen from the ears of a healthy individual were collected and separated by column chromatography. The non-polar lipid fraction was further characterized by gas chromatography—mass spectrometry.

The high lipid concentration of cerumen acts as a physical capture mechanism for pathogens, foreign objects, and debris. Furthermore, a hydrophobic environment impedes the growth and survival of most microorganisms. Cerumen makes the epithelium of the EEC hard to reach and potential receptors for pathogens are covered, thereby preventing the adhesion of these microorganisms. Most research in humans has focused on the characterization and quantification of the lipid content, due to the high hydrophobic feature of cerumen. After lipid extraction of 19 cerumen samples, Bortz et al. reported an average lipid fraction of 52% +/-3% of the dry weight of human cerumen (44). The most recent study on the composition of lipids within human cerumen was performed by Stransky et al. (45), with emphasis on the nonpolar lipid fraction. The results are summarized in Figure 1. Huang et al. used lipid extraction to measure the lipid content and applied thin layer chromatography for the identification of the lipids present in 36 healthy cerumen samples from 20 dogs (46, 47). The total lipid content was on average 49.58% and as such, resembles the lipid content of human cerumen. The following lipids were detected: cholesterol, cholesterol esters, free fatty acids, fatty aldehydes, waxes, triglycerides, lecithin and sphingomyelin. Studies on the general lipid content of equine cerumen were not found.

AD influences the skin and sebum lipid composition. The total skin lipid percentage is significantly lower in human and canine AD patients which includes a decrease in ceramides, important contributors to the skin barrier (48, 49). Gene expression analysis of lesional skin of horses with IBH suggested also an alteration of the ceramide production compared to healthy skin (50). Lesional skin from atopic dogs is defined by a decreased level of sphingomyelins and cholesteryl esters, essential compounds of the epidermal barrier (34). Studies on the lipid composition of the skin and/or sebum in patients with contact dermatitis were not found.

The observed effects of allergic dermatitis on the skin and sebum lipid profile may theoretically have an impact on the cerumen lipid profile and could impair its protective function. Hypothetically, the higher abundance of short chain ceramides in cerumen might result in a more aqueous cerumen, allowing certain microorganisms (such as *Staphylococcus* species and *Malassezia pachydermatis*) to grow and colonize more easily than in normal cerumen and compared to other species present in the ear microbiome, leading to dysbiosis and OE.

The changes of the EEC after insertion of water might resemble the effects of a more aqueous cerumen on certain pathogens. In both dogs and humans, swimming has been associated with a higher risk for developing acute OE (19, 51). The insertion of water in the EEC results in a more humid EEC and an elevation of the pH and leads to maceration of the epidermis. In our opinion, the presence of water within the EEC may impair the function of cerumen thereby creating ideal circumstances for pathogen colonization and, consequently, OE.

#### 3.1.2 Mucins

Mucins are large glycoproteins and the main component of mucus, a viscous secretion lining diverse epithelia. Mucins can be actively secreted into the extracellular space or can be membrane-bound, the latter being released after enzymatic cleavage from the plasma membrane (52, 53). Feig et al. demonstrated the presence of several mucins in human cerumen [secreted mucins: Mucin-5B (MUC5B), Mucin-6 (MUC6), Mucin-7 (MUC7); and membrane-bound mucins: Mucin-1 (MUC1), Mucin-16 (MUC16) and Mucin-20 (MUC20)] (54). Among many other functions, mucins serve as a protective layer against pathogens (54, 55).

Histologic evaluation of the intestines of AD-induced mice showed a lowered content of mucins and was the only attempt made so far on the effect of atopic dermatitis on the expression of mucins (56). Additionally, it was shown that oral *Pediococcus acidilactici* supplementation increased mucin production, but decreased the clinical severity of atopic dermatitis (56). Studying the differences in concentration and structure of certain mucins in the cerumen of allergic patients compared to healthy patients could uncover a new piece of the complex pathogenesis of allergic dermatitis linked to OE and generate possible new insights to disease management.

### 3.2 Antimicrobial molecules present in cerumen

#### 3.2.1 Antimicrobial lipids

Triglycerides are the primary source of free fatty acids after being hydrolyzed by the action of both bacterial lipases and acid lipases of the host’s skin, and possibly earwax too (57). A smaller part is provided by wax esters and cholesterol esters. Within the group of fatty acids (FA) in human cerumen—determined by Stransky et al. as described above—lauric acid (C12:0), myristic acid (C14:0), linoleic acid (C18:2 n-6), sapienic acid (C16:1 n-1) and palmitoleic acid (C16:1 n-7) have been identified. Several studies confirmed the antibacterial activity of these FA to mainly Gram-positive microorganisms, of which an overview is shown in Table 3. Unfortunately, based on the results described by Stransky et al., it was not possible to calculate the exact concentration of the different FA in cerumen. Therefore, it remains unclear whether the different FA in cerumen, as described by Stransky et al., are present at relevant concentrations to inhibit the growth of certain microorganisms.

**Table 3:** Antimicrobial lipids present in cerumen and the minimum inhibitory concentration for skin and/or ear pathogens.

Antimicrobial lipid	Identified in	Minimum inhibitory concentration (MIC)
Lauric acid	Human cerumen (45)	*Cutibacterium acnes*: 3,9 μg/mL (164)
		*Staphylococcus aureus*: 0,97 μg/mL (164)
		*Staphylococcus epidermidis*: 3,9 μg/mL (164)
		*Streptococcus* sp.: 1,00 mg/mL (165)
Linoleic acid	Human cerumen (45), Canine cerumen (60, 158)	*S. aureus*: 3 μg/mL (166)
Sapienic acid	Human cerumen (45)	*S. aureus:* 30 μg/mL (61) *Streptococcus salivarum*: 30 μg/mL (61)
Palmitoleic acid	Human cerumen (45), canine cerumen (158)	*Streptococcus* sp.: 1,27 mg/mL (66)
Oleic acid	Canine cerumen (60, 158)	*S. epidermidis: >* 400 μg/mL (167, 168) *S. aureus:* 400-1000 μg/mL (167, 168)

In addition to FA, the stratum corneum of the skin provides another group of lipids with antimicrobial properties: the sphingosine bases. These are formed enzymatically from the ceramides, present in the intercellular spaces of the stratum corneum. Sphingosine bases have a broad antimicrobial activity against various Gram-positive bacteria and fungi (including *S. aureus, Streptococcus pyogenes, Micrococcus luteus, Cutibacterium acnes, Brevibacterium epidermidis* and *Candida albicans*) (57, 58).

Using gas liquid chromatography and mass spectrometry, the different FA in 36 healthy canine cerumen samples were further characterized, revealing the presence of antimicrobial FA such as myristic acid (C14:0), palmitic acid (C16:0), palmitoleic acid (C16:1), oleic acid (C18:1) and linoleic acid (C18:2) (59). Due to differences in data reporting, it was not possible to compare the fatty acid profile of human and canine cerumen quantitatively.

Another study used gas chromatography to identify the level of total FA within samples of canine cerumen, but these samples were derived from dogs diagnosed with OE (60). Palmitic acid (C16:0), oleic acid (C18:1) and linoleic acid (C18:2) were present in almost all samples (*n* = 95).

Sapienic acid (C16:1n10) was the most abundant FA in human cerumen (45) and is also the major FA present in human sebum (57, 61). In canine cerumen, palmitoleic acid (C16:1n6)—an isomer of sapienic acid—is more commonly identified (46, 62). Both isomers have the ability to inhibit the growth of *S. aureus* (61) but due to the protective effect of staphylococcal oleate hydrolases, *S. aureus* can counter the inhibitory effect of palmitoleic acid, but not of sapienic acid (62). Based on these observations, human sebum and cerumen may be more antimicrobial toward *S. aureus* than canine sebum and cerumen. However, in dogs, the most abundant *Staphylococcus* species on the skin and in the ears is *Staphylococcus pseudintermedius*, and not *S. aureus* (42, 63). Whether palmitoleic acid can inhibit the growth of *S. pseudintermedius* is currently unknown.

Interestingly, oleic acid, identified in canine cerumen in 2 studies as described above (46, 60) was not detected in human earwax in the study by Stranksy et al., even though it was present in human skin sebum as studied by Raghaillaigh et al. (64). Besides its modulatory effect on the immune system, oleic acid is able to increase membrane permeability of *S. aureus* and subsequently induce bacterial death (65, 66). The reason why this FA has been found in canine cerumen samples and not yet in human samples remains unclear, however, more studies with more samples of both species are needed to confirm or invalidate this difference.

Studies describing the FA composition of equine cerumen were not found.

Several studies have already reported alterations in the skin and sebum lipid composition of human Atopic dermatitis patients compared to healthy individuals. By studying the sebum lipid profile of healthy persons and atopic dermatitis patients, a decrease in C18 and C18:1 ceramides and C18 dihydroceramides was observed (67). Furthermore, compared to healthy skin, a decreased concentration of long chain FA and sphingolipids was observed, caused by the effects of IL-4 and IL-13, involved in the inflammatory reaction in atopic dermatitis patients (68).

#### 3.2.2 Antimicrobial proteins

Several researchers investigated the presence of antimicrobial peptides (AMPs) in human, canine and equine cerumen. Until now, the presence of human beta-defensin (hBD)-1, 2, 3; Human Neutrophil Protein (HNP)-1,2,3; LL-37; Secretory leukocyte protease inhibitor (hSLPI); human bactericidal permeability increasing protein (BPI) and two types of immunoglobulins (IgG and IgA) has been confirmed in cerumen. An overview of the different AMPs identified in human, canine and equine cerumen is shown in Table 4. More details on the function, expression and type of these different AMPs can be found in Supplementary Table 2.

**Table 4:** Types of proteins with antimicrobial properties (Antimicrobial Proteins, AMP) identified in the cerumen and EEC of humans, dogs and horses.

AMP	Identified in					
	Human		Canine		Equine	
	Cerumen	EEC	Cerumen	EEC	Cerumen	EEC
*α*-defensin-1	X (69)	–	–	–	–	–
*α*-defensin-2	X (69)	–	–	–	–	–
*α*-defensin-3	X (69)	–	–	–	–	–
*β*-defensin-1	X (69, 169)	X (169–171)	–	–	–	X (172)
*β*-defensin-2	X (69, 169)	X (169–171)	–	–	–	X (172)
*β*-defensin-3	X (69)	–	–	–	–	–
*β*-defensin-3-like	–	–	X (76)	–	–	–
IgA, IgG	–	X (169, 173)	X (158)	–	–	–
Cathelicidins	X (69)	X (170, 171)	X (76)	–	–	–
hSLPI	X (69)	–	–	–	–	–
hBPI	X (69)	–	–	–	–	–
Lactoferrin	X (69)	X (170)	–	–	–	–
Lysozyme	–	X (170)	–	–	–	X (172)

One could question whether the AMPs described above are actively secreted in earwax or whether their presence is just a result of the presence of exfoliated keratinocytes. Schwaab et al. tried to resolve this question by comparing the cell-bound and non-cell-bound fraction of *β*-defensin 1,2,3, hSLPI, hBPI and *α*-defensin-1,2,3. They found that the total concentration of *α*-defensin-1,2,3 before PBS washing of cerumen was significantly higher than the concentration after PBS washing, confirming that the presence of these proteins in earwax is not exclusively due to the presence of keratinocytes (69). Moreover, when looking at the presence of antimicrobial proteins at the skin surface, α-defensins are usually not expressed by keratinocytes (70). Therefore, further investigation is needed to verify whether the presence of *α*-defensins in earwax is due to active secretion by the ceruminous glands. *β*-defensins, LL-37, hSPLI and hBPI are known to be expressed by keratinocytes, so at this point, it is still unclear whether they originate exclusively from the presence of exfoliated keratinocytes in earwax or whether they are actively secreted by ceruminous glands.

The role of AMPs in atopic skin disease was studied frequently in both humans and dogs, retrieving inconsistent results. Depending on the used method, a decreased as well as an increased level of *α*-defensins, *β*-defensins and cathelicidin has been observed in lesional and non-lesional atopic skin (71–76). In normal skin, the expression of *β*-defensin-2, *β*-defensin-3 and cathelicidin is upregulated during infection and inflammation. Besides the current ambiguous effect of AD on the expression of AMPs, one could question why these AMPs—despite their potential impact on the onset of infection—ultimately fail to prevent infection in AD patients, leading to OE.

First, the antipathogenic effects of the AMPs in the EEC could be negligible relative to the change in lipid concentration and composition of cerumen, the impaired skin barrier and the inflammatory parameters involved in allergic skin disease—as discussed above. Moreover, most treatments for CAD will result in a suppression of the AMP-expression, which could theoretically contribute to the relapse of OE in atopic dogs, treated with systemic anti-inflammatory drugs, such as oclacitinib (77), ciclosporin or lokivetmab (78). Furthermore, Santoro et al. reported a defective secretion of *β*-defensin-3 and cathelicidin in non-lesional atopic skin after exposure to pathogens, indicating a loss of function due to an increased adhesion of these AMPs to corneocytes (72). Lastly, an induction of AMP-secretion can hypothetically lead to dysbiosis, as the effect of these host defense peptides can be different for each microorganism present in the EEC. To demonstrate, non-bacterial commensals belonging to the genus of *Malassezia* frequently colonize the EEC of atopic dogs, however, the antipathogenic effect of defensins and cathelicidin on the growth and survival of *Malassezia* species is currently not reported. Moreover, it seems that *Malassezia furfur* and *M. pachydermatis* are able to encourage the secretion of AMPs such as *β*-defensin-2 (79, 80), thereby strengthening the dysbiosis, possibly leading to colonization and infection of the EEC. However, only *M. pachydermatis* is currently associated with OE in atopic dogs, indicating that probably additional factors are needed for colonization.

In conclusion, more studies are necessary to evaluate the effect of allergic dermatitis on the cerumen profile; more specifically on how the changes in different (antimicrobial) compounds can contribute to the development of a more optimal environment for certain pathogens such as *Staphylococcus* species and *Malassezia* species.

## 4 Microorganisms associated with the EEC

### 4.1 Cerumen: barrier or nutrient source for microorganisms

Cerumen is composed of a variety of lipids, proteins and mucins and as such, earwax may potentially serve as an ideal nutrient source for certain bacteria and yeast (81). *M. pachydermatis*, for instance, commonly isolated from otitic ears in dogs, is dependent on exogenous lipid sources due to the lack of a gene coding for fatty acid synthase in its genome (82). Huang et al. and Masuda et al. showed that *M. pachydermatis* can use different ceruminous FA for its growth *in vitro* [myristic acid, palmitic acid, margaric acid, stearic acid, oleic acid and linoleic acid (46, 47, 60)]. As such, cerumen appears to be a good nutrient source for *M. pachydermatis in vitro*. Whether other microorganisms can benefit from the presence of specific components in cerumen for their growth is unclear for now.

However, as discussed above, based on the high lipid content and the presence of both antimicrobial lipids and proteins, the presence of cerumen in the EEC could also complicate the growth and survival of certain microorganisms. Several studies tried to evaluate the effect of cerumen on the *in vitro* growth of bacteria. Campos et al. observed an enhancement of the growth of *S. aureus, Staphylococcus epidermidis, Proteus mirabilis, E. coli, Serratia marcescens and Pseudomonas aeruginosa* by adding a 3% cerumen suspension to bacterial cultures, whereas Chai et al. and Stone and Fulghum observed a decrease in viability of the same bacterial species using the same concentration of cerumen suspension (83–85). However, these studies are hard to compare due to differences in applied culture medium, origin of the samples and sample processing. In a more recent study (86), a cerumen suspension was used to assess the inhibitory effect toward pathogens frequently involved in OE in humans: *S. aureus, P. aeruginosa* and *C. albicans*. Again, both an enhancement (which was the case for 4 out of 31 samples for *S. aureus*; 2 out of 31 samples for *C. albicans* and 27 out of 31 samples for *E. coli*) and an inhibition [which was the case for 27 out of 31 samples for *S. aureus*, 31 out of 31 samples for *P. aeruginosa*, 29 out of 31 samples for *C. albicans* and 4 out of 31 samples for *E. coli(control)*] in growth was observed (86).

Based on the general inhibitory effects of cerumen *in vitro* as shown in the last study, it can be postulated that etiological factors involved in OE (86) intervene with this antimicrobial effect of cerumen, leading to the colonization of pathogens. To demonstrate, the regular insertion of water in the EEC of swimmers is associated with OE (51). Polluted water is a vehicle for certain pathogens such as *P. aeruginosa* and other Gram-negative bacteria (87, 88). Yet, additional factors are needed to allow these pathogens to colonize the EEC. For example, the excessive amount of water into the EEC could—in addition to the maceration of the stratum corneum (51)—generate a mixture with a lower lipid content and a dilution of antimicrobial molecules, thereby facilitating the growth and colonization of pathogens.

Lastly, as cerumen reflects the (patho)physiology of the body with genetic material, lipids, proteins, trace elements, internal and external metabolites reaching earwax from the blood circulation (89), it is not yet clear how the general (patho) physiological state of the host may add to the cerumen’s role as barrier or nutrient source for microorganisms or which specific components may be important.

### 4.2 Studies on the healthy ear microbiome

In the past decades, a few studies tried to identify the microbial community in the healthy EEC of humans, dogs and horses by using culture-dependent methods (26, 39, 59, 90–98). Interestingly, the genera *Staphylococcus, Corynebacterium* and *Bacillus* were frequently identified by culture in different studies in humans, dogs and horses. *Streptococcus* and *Micrococcus* were found in the EEC of both humans and dogs but not in horses. *Malassezia* species were identified in humans (*Malassezia slooffiae, Malassezia sympodialis, M. furfur* and *Malassezia obtusa*), dogs (*M. pachydermatis*) and horses (*Malassezia nana, M. pachydermatis* and *Malassezia globosa*).

More recently, several studies tried to identify the microorganisms present in the healthy canine EEC using next-generation-sequencing (NGS), a culture-independent method (63, 99–107). Most bacteria identified during these studies belong to the following phyla: *Firmicutes, Proteobacteria, Actinobacteria, Bacteroidetes* and *Fusobacteria*. *Ascomycota* and *Basidiomycota* were the most abundant phyla for fungi. A recent study suggests that the microbiome of the canine EEC does not significantly differ from the microbiome of the tympanic bulla microbiome in healthy beagle dogs (108). Four NGS-based studies on the microbial community within the human EEC identified *Firmicutes* and *Actinobacteria* as the most abundant phyla (109–112). Studies on the microorganisms present in the equine EEC based on NGS data are currently lacking.

**Figure 2:**
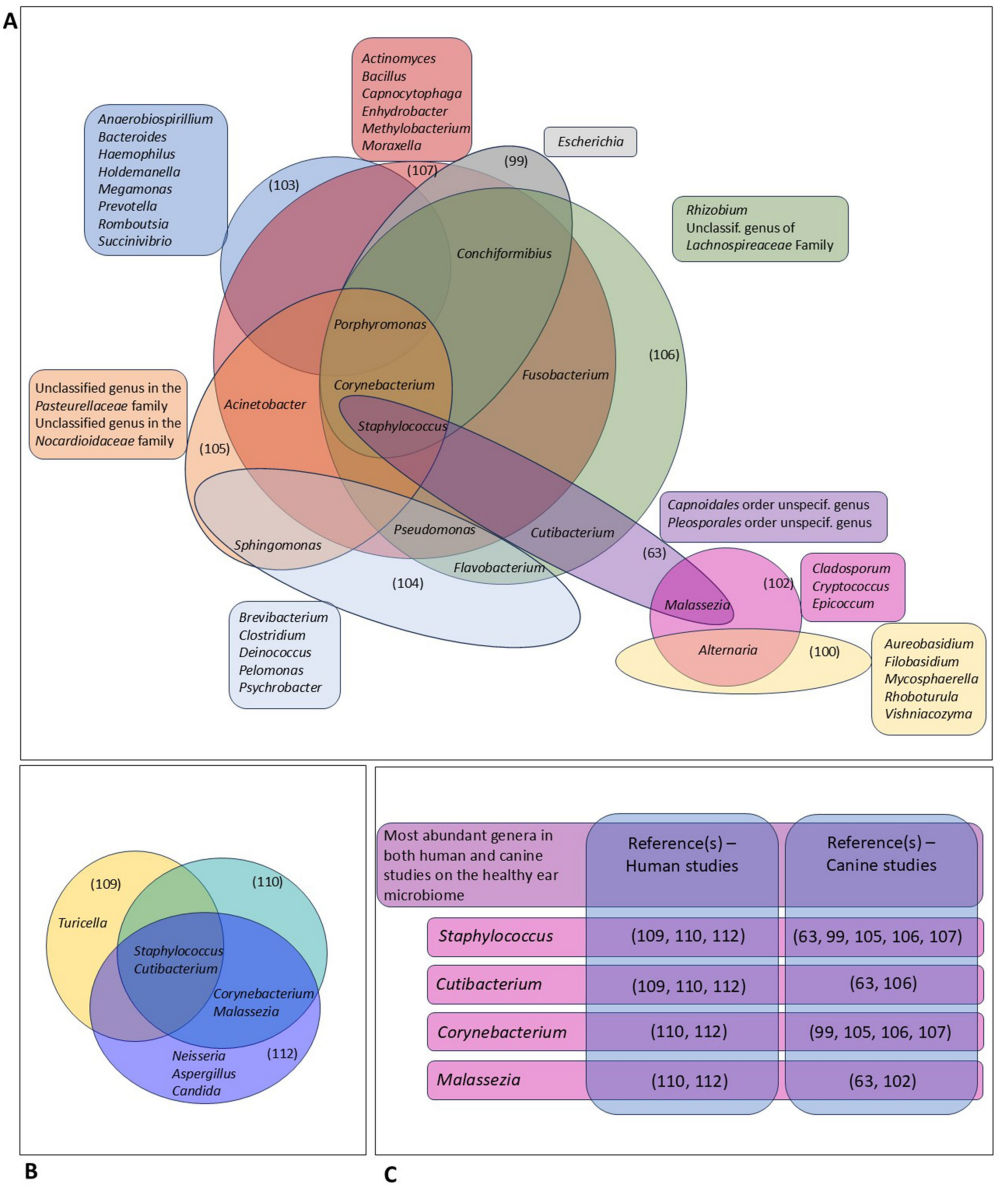
Comparative analysis of different studies on the ear microbiome in dogs and humans using next-generation sequencing (63, 99, 100, 102–107, 109, 110, 112). **(A)** This panel gives an overview of the most abundant genera in different studies on the canine ear microbiome, as reported by the respective authors. Genera that were among the most abundant genera in the respective study, and not in other studies, are displayed outside the diagram. Genera that were among the most abundant genera in at least 2 different studies are included in the diagram. **(B)** Comparison of the most abundant genera identified in 2 different studies on the human ear microbiome. **(C)** This panel shows highly abundant genera identified in both canine and human studies.

In Figure 2, we tried to display the most abundant genera identified in different NGS-based studies on the microbial community in the human and canine EEC, as reported by the respective authors. Yet, as most studies applied different techniques for DNA-extraction, PCR and data processing, this comparison should be interpreted carefully.

In dogs, different genera such as *Alternaria, Acinetobacter, Conchiformibius, Corynebacterium, Flavobacterium, Fusobacterium, Porphyromonas* and *Sphingomonas* were identified in at least 2 different studies. *Pseudomonas, Staphylococcus* and *Malassezia*—common OE-pathogens—were also frequently identified in healthy canine ears, with *Staphylococcus* being described in 5 different studies. Still, many genera were only identified in one single study, which can represent the variations in applied techniques and data reporting, differences in breed and/or environment and living circumstances as well as strong individual variability and/or instability of the community of microorganisms present in the outer ear canal.

The genera *Staphylococcus* and *Cutibacterium* have been identified in 3 NGS-studies describing the human ear microbiome, whereas *Allaiococcus* and *Malassezia* were identified in 2 of the described studies. Interestingly, all genera that were highly abundant in both the human and canine healthy EEC represent facultatively pathogenic bacteria. *Staphylococcus* and *Corynebacterium* species are known OE-pathogens in humans and dogs (see Table 1). However, *Cutibacterium* and *Malassezia* are, so far, only associated with human and canine OE, respectively. In our opinion, species-specific differences in anatomy and physiology of the EEC, the composition of cerumen, underlying conditions as well as microenvironmental factors, may account—at least partly—for the capability of lipophilic *Cutibacterium* to colonize the human and not the canine EEC. The association of the genus *Malassezia* with canine OE only might be related to similar variations, parallel to differences in pathogenic features of the dog specific *M. pachydermatis*, compared to the *Malassezia* species identified in human ear canals.

Based on present data, it may be interesting to consider how these bacteria and other microorganisms are able to survive and grow in the microenvironment of the EEC. In other words, which of the microorganisms identified in the studies described above, are just passing by and which species have specifically developed certain strategies to survive, grow, and even colonize in this microenvironment? Recently, the interest in the human and canine ear microbiome is increasing and new, culture-independent methods offer the ability to obtain a more thorough understanding of this microbiome. However, NGS cannot easily distinguish between viable and dead microorganisms, as it is based on the presence of bacterial DNA at a certain sampling moment. Moreover, the studies described above used different techniques for sample collection, DNA extraction and data analysis, which makes it hard to compare the results. Ear samples may—similar to skin samples—be considered as low-biomass samples, which require caution during processing to avoid contamination. Recent recommendations for obtaining reliable results using 16S rRNA sequencing on low-biomass-samples suggest a minimal concentration of 10^6^ bacteria per sample and an improved protocol for DNA extraction and PCR (113). Unfortunately—to the best of our knowledge—no quantitative data are available on the total number of bacteria present in the ear samples analyzed by NGS in the different studies investigating the ear microbiome.

Lastly, until now, no information is available yet on the microbiome-host interactions and the potential benefits of the presence of a microbiome in the EEC. We conclude that—at this moment—the existence, complexity and stability of an ear microbiome has not been sufficiently demonstrated in available studies and needs further research.

### 4.3 Effect of allergic dermatitis on the microorganisms in the EEC

The skin of atopic humans is defined by a decrease in bacterial diversity, richness and evenness and a change in skin microbiome composition, with higher abundances of *Staphylococcus* species (112, 114) and *Cutibacterium* species (112). The use of artificial intelligence and machine learning may aid in the interpretation of complex metagenomics and even transcriptomics data to differentiate between healthy and atopic dermatitis patients (115). Whether this dysbiosis is a cause or result of atopic dermatitis is currently unknown. As the pathogenesis of human and CAD is very similar (116), CAD can potentially affect the colonization capabilities of facultative pathogens in the EEC as well. A few studies tried to compare the microbial composition and diversity of the EEC of healthy and atopic dogs (99, 102, 104), however, a significant decrease in microbial diversity in the ears of atopic dogs compared to healthy dogs was not demonstrated so far. Nonetheless, some interesting differences between healthy and atopic ears were observed, such as an increase in the abundance of the genera *Staphylococcus, Sphingomonas, Ralstonia, Methylotenera, Lactobacillus, Hymenobacter, Chryseobacterium, Alcaligenes and Malassezia* and a decrease in the abundance of the genera *Macrococcus, Brevibacterium* and *Escherichia* in the EEC of atopic dogs compared to healthy individuals. It was recently described that 16S amplicon profiling appears to be a more sensitive method to describe bacterial populations in canine otitis cases compared with conventional culture-dependent methods (117).

Studies on the effect of CD in humans and IBH in horses on the microbial communities in the EEC of these species are currently lacking.

## 5 The development of allergic dermatitis and OE: impact of neonatal events and the gut and skin microbiome

Although one of the main topics of the current review is the effect of the characteristic changes of AD on the ear microbiome to better understand the development of OE in AD patients, it is intriguing to discuss another possible correlation between the microbiome, allergic disease and OE. Recent insights point toward the importance of the early-life (gut) microbiome as an important factor in the development of allergic disease in humans (12, 118, 119) and dogs and cats (120).

Without going into detail, it has been suggested that prenatal and early-life postnatal events that may interfere with the establishment of the child’s microbiome, such as diet, antimicrobial exposure, environmental bacterial load and early-life cytomegalovirus infection, may increase the risk of allergic dermatitis (121–125) and even otitis media (126) in these children or dogs (127). It was recently even suggested that artificial intelligence-supported analysis of the gut microbiota may be used to accurately diagnose atopic dermatitis in humans (115).

In addition, the auditory-gut-brain axis has very recently become a topic of research interest, suggesting that connections have been found between the external ear, the central auditory system and the gut microbiome (128). An interesting study found that colonization of the gut with *Candida albicans* in mice was associated with the presence of pro-inflammatory cytokines in the ear (129). Furthermore, inflammatory bowel disease in humans is associated with hearing loss in autoimmune inner ear disease and Cogan’s syndrome (130).

To our knowledge, the correlation between OE and prenatal/early postnatal disruption of the skin, ear or gut microbiome has not yet been studied in humans or animals, but could be included in future epidemiologic surveys to further understand the risk factors associated with the development of OE.

## 6 The effect of OE prevention and treatment on the ear microbiome

While allergic dermatitis and the presence of OE can have a significant effect on the microbial community of the EEC, also preventive interventions or OE treatments, often including the use of agents with antimicrobial properties (8, 131), may affect the ear microbiome. Aqueous canine otic treatment products aim to approximate the pH range of a healthy dog’s ear in order to be less likely to disrupt the micro-environment of the EEC (132) or may even be used to re-acidify it (133). Preventive use of a topical anti-inflammatory glucocorticoid in atopic dogs without clinical signs of otitis does not seem to affect the ear canal microbiota and mycobiota (134). Treatment of dogs with OE with an anti-inflammatory pomegranate otic treatment mainly had an effect on the mycobiome with markedly lowered *M. pachydermatis* presence after treatment (135). Chermprapai et al. (136) showed that a topical antimicrobial treatment increased the diversity of bacterial and fungal compositions in course of time on both AD and healthy skin in dogs. Matsui et al. (137) showed that doxycycline not only inhibited *Staphylococcus aureus* strains isolated from skin lesions of patients with atopic dermatitis in a mouse model, but also had a strong inhibitory effect on epidermal Langerhans cells and Th2 cell development, thereby suppressing the acute inflammation.

Even though probiotics are currently being considered for the prevention of otitis media in human medicine, no similar attempts have yet been reported in either human or veterinary medicine for otitis externa to the best of our knowledge (138–140). On the other hand, the microbiome has been suggested as a potential early-life target to prevent the development of allergic dermatitis and may therefore also indirectly add to the prevention of AD-related complications, such as otitis externa (12).

## 7 Discussion

This review provides a cross-species comparison of the current knowledge on the EEC, revealing structural and physiological differences between humans, dogs and horses. The relevance of these species-specific differences is highlighted by the example of atopic dermatitis, which manifests differently in humans and dogs, despite a similar pathophysiology. In humans, atopic dermatitis shows a low prevalence of otitis externa (OE), whereas in dogs, it shows a high prevalence. This review attempts to explain this difference in predisposition by identifying differences in anatomy, lipid composition and differences in the composition of the potential ear microbiome (141).

Additionally, this review compiles, for the first time to the author’s knowledge, studies on the molecular composition of cerumen in humans, dogs and horses, identifying interesting species-specific differences. Cerumen is known to trap microorganisms and protect the epithelium, thereby preventing adhesion and exerting direct antimicrobial effects through molecules such as oleic acid and antimicrobial proteins. Together, these findings indicate that the EEC creates a hostile environment for microbes across all observed host species.

Given the different prevalence of allergy-induced OE in at least three mammalian species, this review aimed to identify key factors in the development of OE in patients with allergic dermatitis. In cases of OE, certain pathogens, such as *Staphylococcus* and *Malassezia* species, manage to survive and multiply within the hostile environment of the EEC, as various. It is hypothesized that in allergic dermatitis-related OE, the pathophysiological processes may counteract the antimicrobial properties of the EEC. Studies suggest that allergic dermatitis impacts skin parameters such as pH, lipid content, and the presence of antimicrobial molecules. Nevertheless, more research is needed to understand the effect of allergic dermatitis on the EEC and to unravel the link between allergic dermatitis and OE.

Recently, there has been increasing interest in investigating the existence of an ear microbiome, suggesting that microorganisms can also survive and multiply in a healthy ear canal. Currently, little evidence exists on which species can counter the antimicrobial features of cerumen and form a stable, balanced ear microbiome. Although inter-and intra-species comparisons of available studies on the ear microbiome are challenging, it appears that the few species identified across most studies are facultative pathogenic micro-organisms associated with ear infections. Considering the low biomass of ear samples and the associated challenges related to appropriate experimental set-up and interpretation of results, there is still a need for high quality ear microbiome studies both in human and veterinary medicine.

The microbiome has been suggested as a potential target for treatment and prevention of OE. However, the impact of the microbiome—including the ear microbiome and the gut microbiome (“auditory-gut-brain axis”)—on the development and persistence of ear infections such as OE, otitis media, and otitis interna requires further investigation.

In conclusion, this review identifies several gaps in the research on the unique environment within the EEC in three mammalian species with known allergic dermatitis-related prevalence of OE. More research, combining known and yet-to-be-explored processes in different species using a ‘One Health’ approach, could provide exciting new insights. These insights may lead to improved prevention and therapeutic options in both human and veterinary medicine.
