# Supplementary material for: The role of the microbiome in allergic dermatitis-related otitis externa: a multi-species comparative review
Source: Front Vet Sci. 2024 Dec 16;11:1413684. doi: 10.3389/fvets.2024.1413684 (PMC11683847; doi:10.3389/fvets.2024.1413684)
Supplement: Supplementary file 1 [file Table_1.docx]

|  | **Allergic contact dermatitis (CD) in humans** | **Canine Atopic dermatitis (CAD) in dogs** | **Insect bite hypersensitivity (IBH)**  **in horses** |
| --- | --- | --- | --- |
| **Definition** | Allergic CD is a **local pruritogenic inflammation** of the epidermis after regular contact with allergens (1) | CAD is a **generalized inflammatory skin reaction** induced after multiple contact with environmental allergens, causing an Ig-E mediated immune respons (2) | IBH is a **generalized skin reaction** caused by combination of a type I and type IV hypersensitivity against salivary antigens of C*ulicoides* and other insects (3) |
| **Chemokines and cytokines associated with disease** | IL-1β, IL-1Ra, IL-6, IL-8, IL-36α, IL-36β, IL-33 (1,4) | Sensitization: IL-4, IL-13 (5);  Reexposure after sensitization: TNF-α, histamine, serotonin, substance P, IL-8, IL-10, IL-31 (5–7);  Chronic phase: IFN-γ (7) | Histamine, leukotrienes, IL-13 (8) |
| **Immune cells involved in the pathogenesis** | Langerhans' cells, CD8+ Tc1/Tc17 and CD4+ Th1/Th17 cells (1,4) | Sensitization: Langerhans' cell, naïve T cell, B cell (5);  Reexposure after sensitization: Th2-cell, plasmacell, mastcell (5);  Chronic phase: Th1-cell (7) | Th2-cell (8) |
| **Allergens associated with OE** | Plastic or metal devices (e.g. hearing aids); chemicals in cosmetics, shampoos and detergentia or ingredients in ototopical products after regular use (e.g. benzalkonium choride, neomycin, gentamycin) (9) | Environmental allergens (pollen, grass, house dust mites, food components, etc…) (10) | Salivary antigens from *Culicoides* insects (8) |
| **Prevalence of allergic OE** | Allergic dermatitis was identified as the underlying cause in **23-59%** of the patients with OE, according to different studies (9) | In one study, **8%** of all dogs suffering from OE was diagnosed with AD (11). Further on, 48-60% of the patients with AD suffer from OE (12) | No exact numbers available, but OE is considered as a rare condition in horses and the EEC is only occasionally involved in horses who suffer from IBH (8,13,14) |

**Supplementary table 1.** Pathogenic features of allergic diseases associated with OE in humans, dogs and horses

**References**

1. Martin S, Esser P, Weber F, Jakob T, Freudenberg M, Schmidt M, Goebeler M. Mechanisms of chemical-induced innate immunity in allergic contact dermatitis. *Allergy* (2011) 66:1152–1163. doi: 10.1111/J.1398-9995.2011.02652.X

2. Halliwell R. Revised nomenclature for veterinary allergy. *Vet Immunol Immunopathol* (2006) 114:207–208. doi: 10.1016/j.vetimm.2006.08.013

3. Kurotaki T, Narayama K, Oyamada T, Yoshikawa H, Yoshikawa T. Immunopathological study on equine insect hypersensitivity (“kasen”) in Japan. *J Comp Pathol* (1994) 110:145–152. doi: 10.1016/S0021-9975(08)80186-7

4. Mattii M, Ayala F, Balato N, Filotico R, Lembo S, Schiattarella M, Patruno C, Marone G, Balato A. The balance between pro- and anti-inflammatory cytokines is crucial in human allergic contact dermatitis pathogenesis: the role of IL-1 family members. *Exp Dermatol* (2013) 22:813–819. doi: 10.1111/EXD.12272

5. Marsella R, Sousa CA, Gonzales AJ, Fadok VA. Current understanding of the pathophysiologic mechanisms of canine atopic dermatitis. *J Am Vet Med Assoc* (2012) 241:194–207. doi: 10.2460/javma.241.2.194

6. Lecru L-A, Combarros D, Moog F, Marinovic L, Kondratjeva J, Amalric N, Pressanti C, Cadiergues MC. Multiplex Cytokine Analyses in Ear Canals of Dogs Suggest Involvement of IL-8 Chemokine in Atopic Otitis and Otodectic Mange-Preliminary Results. *Animals (Basel)* (2022) 12:575. doi: 10.3390/ANI12050575

7. McCandless EE, Rugg CA, Fici GJ, Messamore JE, Aleo MM, Gonzales AJ. Allergen-induced production of IL-31 by canine Th2 cells and identification of immune, skin, and neuronal target cells. *Vet Immunol Immunopathol* (2014) 157:42–48. doi: 10.1016/j.vetimm.2013.10.017

8. Schaffartzik A, Hamza E, Janda J, Crameri R, Marti E, Rhyner C. Equine insect bite hypersensitivity: What do we know? *Vet Immunol Immunopathol* (2012) 147:113–126. doi: 10.1016/j.vetimm.2012.03.017

9. Sood S, Strachan DR, Tsikoudas A, Stables GI. Allergic otitis externa. *Clin Otolaryngol Allied Sci* (2002) 27:233–236. doi: 10.1046/j.1365-2273.2002.00584.x

10. Zur G, Ihrke PJ, White SD, Kass PH. Canine atopic dermatitis: a retrospective study of 266 cases examined at the University of California, Davis, 1992-1998. Part I. Clinical features and allergy testing results. *Vet Dermatol* (2002) 13:89–102. http://www.ncbi.nlm.nih.gov/pubmed/11972892 [Accessed April 3, 2018]

11. Saridomichelakis MN, Farmaki R, Leontides LS, Koutinas AF. Aetiology of canine otitis externa: a retrospective study of 100 cases. *Vet Dermatol* (2007) 18:341–347. doi: 10.1111/j.1365-3164.2007.00619.x

12. Bizikova P, Santoro D, Marsella R, Nuttall T, Eisenschenk MNC, Pucheu-Haston CM. Review: Clinical and histological manifestations of canine atopic dermatitis. *Vet Dermatol* (2015) 26:79-e24. doi: 10.1111/vde.12196

13. Peeters L, Verlinden T, Brebels M, Buys N, Janssens S. Environmental factors affecting the prevalence of insect bite hypersensitivity in Belgian warmblood horses in Vlaanderen. *Commun Agric Appl Biol Sci* (2011) 76:205–9.

14. Odelros E, Kendall A, Wulcan S, Bergvall K. Otitis externa in eight horses – clinical signs, treatment and prognosis. *Vet Dermatol* (2019) 30:430. doi: 10.1111/vde.12763
