# Supplementary material for: The role of the microbiome in allergic dermatitis-related otitis externa: a multi-species comparative review
Source: Front Vet Sci. 2024 Dec 16;11:1413684. doi: 10.3389/fvets.2024.1413684 (PMC11683847; doi:10.3389/fvets.2024.1413684)
Supplement: Supplementary file 2 [file Table_2.docx]

**Supplementary Table 2.** Characteristics of proteins with antimicrobial activities found in cerumen and/ or the EEC in different species

|  | **Isolated from/ identified in** | | **Expression/secretion in the EEC** | **Involved in** | **Function** |
| --- | --- | --- | --- | --- | --- |
| **DEFENSINS** | | | | | |
| **α-defensin-1**  **α-defensin-2**  **α-defensin-3** | Human cerumen(1) | | Secreted actively by the glandular tissue | **Antimicrobial activities and aspecific immune response** | - Neutralization of pathogens (e.g. Human Immunodeficiency Virus (HIV), Herpes Simplex Virus (HSV) and Influenzavirus (2) - Chemoattraction of monocytes, T-cells and dendritic cells in inflammatory reactions (3–5) |
| **β-defensin-1** | Human cerumen (1,6) | The human EEC (6–8) and the equine EEC (9) | Expressed by the epithelial cells and secreted actively by the glandular tissue | **Antimicrobial activities and aspecific immune response** | - Interaction with the bacterial membrane leading to the formation of pores, membrane disruption and lysis of the microorganism (3–5) - Chemoattraction of monocytes, T-cells and dendritic cells in inflammatory reactions (3–5) |
| **β-defensin-2** | Human cerumen (1,6) | The human EEC (6–8) and equine EEC (9) |  |  |  |
| **β-defensin-3** | Human cerumen (1) | |  |  |  |
| **β-defensin-3-like** | Canine cerumen (10) | | NA | **NA** | NA |
| **IMMUNOGLOBULINS** | | | | | |
| **IgA, IgG** | Canine cerumen (11) | The human EEC (6,12) | Expressed by the epithelial cells and immune cells in the dermis | **Specific immune response** | - Antigen-binding |
| **CATHELICIDINS** | | | | | |
| **LL37, cCath** | Human cerumen  (1)  Canine cerumen (13) | The human EEC (7,8) | Secreted actively by the ceruminous glands | **Antimicrobial activities, aspecific and specific immune response** | - Antibacterial activity: disruption of the gram-positive and gram-negative bacterial membrane and inhibition of their protein and/or RNA synthesis (14) - Chemoattractant of neutrophils, monocytes and T-cells (14) - Activation of mast cells (14) |
| **OTHER PROTEINS** | | | | | |
| **hSLPI** | Human cerumen (1) | | Expressed by the epithelial cells | **Antimicrobial activities** | - Protective factor against endogenous proteases (15) - Broad-spectrum antibacterial activity against a variety of micro-organisms including *S. aureus, Pseudomonas aeruginosa* and *Aspergillus fumigatus* (15) |
| **hBPI** | Human cerumen (1) | | Expressed by the epithelial cells | **Antimicrobial activities** | - Antibacterial activity: interaction with the gram-negative bacterial membrane, which leads to an increase in membrane permeability and bacterial death (16) |
| **Lactoferrin** | Human cerumen (1) | The human EEC (7) | Secreted actively by the ceruminous glands | **Antimicrobial activities** | - Bacteriostatic and bactericidal mode of action: selective binding of the Fe^3+^ ion leading to a reduced availability of this nutrient for bacteria and interaction with the bacterial membrane of gram-positive and gram-negative bacteria (17) |
| **Lysozyme** | The human EEC (7) and equine EEC (9) | | Secreted actively by the ceruminous glands | **Antimicrobial activities and aspecific immune response** | - Antibacterial activity: interaction with the bacterial membrane and hydrolysis of peptidoglycan, which results in the formation of pores and killing of bacteria (18) - Modulation of the innate host defense: interaction with the pro-inflammatory immune response (18) |

**References:**

1. Schwaab M, Gurr A, Neumann A, Dazert S, Minovi A. Human antimicrobial proteins in ear wax. *European Journal of Clinical Microbiology and Infectious Diseases* (2011) 30:997–1004. doi: 10.1007/s10096-011-1185-2

2. Lehrer R, Lu W. α-defensins in human innate immunity. *Immunol Rev* (2012) 245:84–112. doi: 10.1111/j.1600-065X.2011.01082.x.

3. Ganz T. Defensins: Antimicrobial peptides of innate immunity. *Nat Rev Immunol* (2003) 3:710–720. doi: 10.1038/nri1180

4. Klotman ME, Chang TL. Defensins in innate antiviral immunity. *Nat Rev Immunol* (2006) 6:447–456. doi: 10.1038/nri1860

5. Gallo RL, Hooper L V. Epithelial antimicrobial defence of the skin and intestine. *Nat Rev Immunol* (2012) 12:503–516. doi: 10.1038/nri3228

6. Yoon JY, Park WJ, Lee JE. Presence of hBD-1 and hBD-2 in human cerumen and external auditory canal skin. *Acta Otolaryngol* (2008) 128:871–875. doi: 10.1080/00016480701785020

7. Stoeckelhuber M, Matthias C, Andratschke M, Stoeckelhuber BM, Koehler C, Herzmann S, Sulz A, Welsch U. Human ceruminous gland: ultrastructure and histochemical analysis of antimicrobial and cytoskeletal components. *Anat Rec A Discov Mol Cell Evol Biol* (2006) 288:877–84. doi: 10.1002/ar.a.20356

8. Yoon YJ, Lee EJ. Spatial distribution of antimicrobial peptides and mast cells in the skin of the external auditory canal. *Journal of Laryngology and Otology* (2011) 125:1–5. doi: 10.1017/S0022215111002271

9. Yasui T, Tsukise A, Fukui K, Kuwahara Y, Meyer W. Aspects of glycoconjugate production and lysozyme- and defensins-expression of the ceruminous glands of the horse (Equus przewalskii f. dom.). *Eur J Morphol* (2005) 42:127–134. doi: 10.1080/09243860500202507

10. Santoro D. Comparison of the quantity and antimicrobial activity of host defence peptides in ear canals between healthy and atopic dogs: A preliminary study. *Vet Dermatol* (2023) 34:452–459. doi: 10.1111/VDE.13164

11. Huang H-P. Studies of the microenvironment and microflora of the canine external ear canal - Theses. Glasgow: University of Glasgow. (1993). https://theses.gla.ac.uk/678/ [Accessed June 14, 2022]

12. Sirigu P, Cossu M, Puxeddu P, Marchisio A, Perra M. Human ceruminous glands: a histochemical study. *Basic Appl Histochem* (1983) 24:257–265.

13. Santoro D. Comparison of the quantity and antimicrobial activity of host defence peptides in ear canals between healthy and atopic dogs: A preliminary study. *Vet Dermatol* (2023) 34:452–459. doi: 10.1111/VDE.13164

14. Bals R, Wilson JM. Cathelicidins - A family of multifunctional antimicrobial peptides. *Cellular and Molecular Life Sciences* (2003) 60:711–720. doi: 10.1007/s00018-003-2186-9

15. Sallenave JM. Secretory leukocyte protease inhibitor and elafin/trappin-2: Versatile mucosal antimicrobials and regulators of immunity. *Am J Respir Cell Mol Biol* (2010) 42:635–643. doi: 10.1165/rcmb.2010-0095RT

16. Elsbach P, Weiss J. Role of the bactericidal/permeability-increasing protein in host defence. *Curr Opin Immunol* (1998) 10:45–49. doi: 10.1016/S0952-7915(98)80030-7

17. Hassoun LA, Sivamani RK. A systematic review of lactoferrin use in dermatology. *Crit Rev Food Sci Nutr* (2017) 57:3632–3639. doi: 10.1080/10408398.2015.1137859

18. Ragland SA, Criss AK. From bacterial killing to immune modulation: Recent insights into the functions of lysozyme. *PLoS Pathog* (2017) 13:e1006512. doi: 10.1371/journal.ppat.1006512
